# Epigenetic and transcriptional responses underlying mangrove adaptation to UV-B

**DOI:** 10.1016/j.isci.2021.103148

**Published:** 2021-09-20

**Authors:** Yushuai Wang, Chenglong Huang, Weishun Zeng, Tianyuan Zhang, Cairong Zhong, Shulin Deng, Tian Tang

**Affiliations:** 1 State Key Laboratory of Biocontrol and Guangdong Key Laboratory of Plant Resources, School of Life Sciences, Sun Yat-sen University, Guangzhou 510275, Guangdong, People’s Republic of China; 2 Hainan Academy of Forestry (Hainan Academy of Mangrove), Haikou 571100, Hainan, People’s Republic of China; 3 CAS Key Laboratory of South China Agricultural Plant Molecular Analysis and Genetic Improvement & Guangdong Provincial Key Laboratory of Applied Botany, South China Botanical Garden, Chinese Academy of Sciences, Guangzhou 510650, People’s Republic of China; 4 Xiaoliang Research Station for Tropical Coastal Ecosystems, South China Botanical Garden, Chinese Academy of Sciences, Guangzhou 510650, People’s Republic of China

**Keywords:** molecular biology, plant biology, transcriptomics

## Abstract

Tropical plants have adapted to strong solar ultraviolet (UV) radiation. Here we compare molecular responses of two tropical mangroves *Avecennia marina* and *Rhizophora apiculata* to high-dose UV-B. Whole-genome bisulfate sequencing indicates that high UV-B induced comparable hyper- or hypo-methylation in three sequence contexts (CG, CHG, and CHH, where H refers to A, T, or C) in *A. marina* but mainly CHG hypomethylation in *R. apiculata*. RNA and small RNA sequencing reveals UV-B induced relaxation of transposable element (TE) silencing together with up-regulation of TE-adjacent genes in *R. apiculata* but not in *A. marina*. Despite conserved upregulation of flavonoid biosynthesis and downregulation of photosynthesis genes caused by high UV-B, *A. marina* specifically upregulated ABC transporter and ubiquinone biosynthesis genes that are known to be protective against UV-B-induced damage. Our results point to divergent responses underlying plant UV-B adaptation at both the epigenetic and transcriptional level.

## Highlights

• *Avicennia* and *Rhizophora* mangroves have adapted to high UV radiation
• UV-B leads to divergent epigenetic responses between *A. marina* and *R. apiculata*
• UV-B induces TE transcriptional de-repression in *R. apiculata,* not in *A. marina*
• Similarity and difference in pathways underlie the adaptation of mangroves to UV-B

## Graphical abstract

**Figure:**
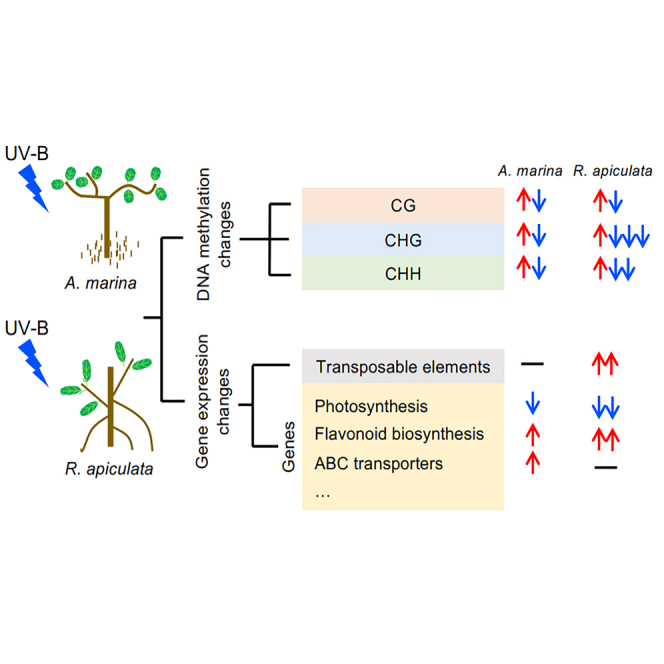

## Introduction

Plants are constantly exposed to sunlight and affected by solar radiation. The ultraviolet-B (UV-B, 280–320 nm) comonent of sunlight can cause diverse responses in plants depending on its fluence rate, duration, and wavelength (Brown and Jenkins, 2008; Jenkins, 2009). Low doses of UV-B initiate UV-B-specific signaling, induce photomorphogensis (Ulm and Nagy, 2005; Tong et al., 2008; Jansen and Bornman, 2012; O'Hara et al., 2019), and promote the synthesis of photoprotective secondary metabolites, including the UV-absorbing flavonoids and anthocyanins (Yin and Ulm, 2017; Del Valle et al., 2020; Shamala et al., 2020). These responses are mediated by the UV-B specific photoreceptor UV RESISTANCE LOCUS 8 (UVR8) (Brown et al., 2005; Kaiserli and Jenkins, 2007; Jenkins, 2009; Rizzini et al., 2011; Liang et al., 2019). High doses of UV-B also cause cellular damage to DNA, RNA, proteins, and lipids (Hollosy, 2002; Nawkar et al., 2013). The adverse effects of UV-B often involve the production of reactive oxygen species (ROS) and the activation of nonspecific stress signaling pathways (Tossi et al., 2012; Hideg et al., 2013), including DNA damage and wound/defense signaling molecules (Stratmann, 2003; Jenkins, 2009; Vanhaelewyn et al., 2016). Photosynthesis is particularly sensitive to UV-B radiation, with photosystem II (PSII) more vulnerable to UV-B than photosystem I (Hollosy, 2002; Wilson and Ruban, 2019). In general, chronic UV-B radiation activates acclimation responses while acute exposure has a more severe effect (Boyko et al., 2006; Lake et al., 2009; Kataria et al., 2014).

Although transcriptome responses triggered by UV-B radiation have been extensively studied in plants (Frohnmeyer and Staiger, 2003; Vanhaelewyn et al., 2016; Wang et al., 2019; Qian et al., 2020), relatively little is known about plant epigenetic effects of this radiation (Casati et al., 2006, 2008). Current knowledge is largely based on the genetic analyses of a few loci. In *Arabidopsis*, UV-B stress mediates release of transgene silencing. The resulting transcriptional reactivation correlated with alterations in histone occupancy and acetylation but not with prominent changes in cytosine methylation (Lang-Mladek et al., 2010). In contrast, UV-B has been reported to induce dynamic DNA methylation at specific genes or transposable elements (TEs) in other species, including hypomethylation in maize (Rius et al., 2016), Norway spruce (Ohlsson et al., 2013), and *Artemisia annua* (Pandey and Pandey-Rai, 2015), and hypermethylation in grapevine (Marfil et al., 2019). Interestingly, DNA methylation appears to play a potential role in adaptation to high UV-B irradiation. The maize R2R3-MYB transcription factor P1 involved in activation of flavonoid biosynthesis was demethylated in response to UV-B and is expressed higher in a high-latitude than in a low-latitude landrace (Czemmel et al., 2009; Rius et al., 2016). Recently, Jiang et al. (Jiang et al., 2021) reported that direct interaction between UVR8 and *de novo* DNA methyltransferase DOMAINS REARRANGED METHYLTRANSFERASE 2 (DMR2) is critical for UV-B-induced DNA methylation alteration and transcriptional de-repression in *Arabidospis*, suggesting UV-B-mediated methylation changes are prevalent in plants. However, the genome-wide methylation pattern in response to UV-B radiation and its potential impact on the whole transcriptome during plant adaptation to changing environments remain poorly understood.

Tropical plants receive higher UV radiation than plants inhabiting temperate regions (Frederick, 1989). Ozone depletion may have further increased plant UV exposure at all latitudes over the past few decades (Searles et al., 2001; Austin and Wilson, 2006; Caldwell et al., 2007; Bornman et al., 2019). Thriving in the dynamic tropical and subtropical intertidal zones, mangroves often have thick, succulent leaves that can increase the attenuation of UV radiation. These tree species represent a good system to understand the diverse mechanisms underlying plant UV adaptation. Previous studies have shown that UV-absorbing phenolic compounds form a UV-screen in the epidermis of mangrove leaves (Lovelock et al., 1992). Compared with other tropical forest plants, mangroves exhibit particularly high beta carotene content in sun leaves, which may play a photoprotective role (Krause et al., 2003; D'Alessandro and Havaux, 2019). Nevertheless, the concentration of UV-absorbing compounds varies between mangrove species, depending on sampling sites, seasons, and genetic variation (Lovelock et al., 1992; Kathiresan, 2018). Different mangrove species also vary in their response to UV treatment (Lovelock et al., 1992). For example, *Brugiera gymnorrhiza* and *Rhizophora apiculata* possess similar levels of UV-absorbing compounds whereas *R. apiculata* shows no change in total chlorophyll contents in response to UV radiation, probably due to its greater carotenoid concentration and greater succulence (Lovelock et al., 1992). Molecular mechanisms underlying mangrove responses to harsh UV exposure are largely unknown. Such knowledge will improve our understanding of how plants adapt to high fluxes of UV radiation during long-term evolution.

*Avecennia marina* (Acanthaceae) and *Rhizophora apiculata* (Rhizophoraceae), diverged about 120 Mya, are the two most common and widespread true mangroves with available whole-genome sequences (Xu et al., 2017). These two species have adapted to strong UV-B radiation in tropical coastlines for millions of years while their differing leaf anatomical characters suggest that molecular mechanisms underlying their adaptation to UV-B radiation may be different. The leaves of *A. marina* are slightly hairy and scattered with salt glands, while *R. apiculata* leaves are smooth and succulent (Poompozhil and Kumarasamy, 2014). As epigenetics allows individuals to quickly explore an adaptation to environmental change, these mangrove species may exhibit different epigenetic responses to UV-B given their differences in leaf anatomy. Such epigenetic responses may further induce differential expression of TEs or genes involved in UV-B adaptation. While physiological reponse of *A. marina* to UV-B remains unclear, accumulation of UV-B absorbing compounds has been reported in the congeneric species *A. germinans* (Wingfield et al., 2017). Both UV-B absorbing compounds and photosynthetic pigments are known to perform a photoprotective function in *R. apiculata* (Lovelock et al., 1992).

Aiming to understand the diversifying strategies underlying mangrove UV-B adaptation, we compared epigenetic and transcriptional responses of *A. marina* and *R. apiculata* to UV-B treatment using methylome, transcriptome, and small RNA profiling of their leaves. We found that high UV-B leads to divergent epigenetic responses between *A. marina* and *R. apiculata*, including TE de-repression in *R. apiculata*. Furthermore, the *A. marina* transcriptome was more stable under UV-B exposure than the *R. Apiculata* transcriptome. We inferred the key pathways that potentially confer UV-B adaptation by differential expression analyses. We also explored the association between epigenetic and gene expression changes induced by UV-B.

## Results

### UV-B induces widespread non-CG DNA hypomethylation in *R. apiculata* but not in *A. marina*

To determine the methylation changes in *A. marina* and *R. apiculata* genomes in response to UV-B exposure, seedlings of each species were irradiated with or without additional UV-B (∼92.6 μW/cm^2^; hereafter as “treated” and “control”, respectively) for eight hours per day for seven days. No obvious stress symptoms were observed in either species after the UV-B treatment (Figure S1). Physiological analyses of chlorophyll a, chlorophyll b, and flavonoid content in leaves of UV-B treated and control plants from each species only detected a significant reduction in chlorophyll a by 34.8% in *R. apiculata* (two-tailed t test, p < 0.05, Figure S2). Leaves of UV-B-treated and control plants were harvested and used for whole-genome bisulfite sequencing (BS-seq), each with three biological replicates (Table S1). On average, BS-seq covered more than 80% of all cytosines (including CG, CHG, and CHH, where H corresponds A, T, or C) in the genomes of *A. marina* and *R. apiculata* with sequencing depths of 16.6× and 29.2×, respectively (Table S1). Coverages of the UV-B and control *R. apiculata* (91.6 ± 1.1% vs. 92.3 ± 1.0%, two-tailed t test, p > 0.05) was comparable, but slightly lower in the treated than control *A. marina* plants (81.1 ± 4.6% vs. 88.2 ± 1.8%; two-tailed t test, p < 0.001). Sliding window analysis of methylation levels revealed good reproducibility of biological replicates in all three sequence contexts (Pearson’s correlation, all *cor* > 0.99; Figure S3), except that one replicate of UV-B-treated *R. apiculata* showed relatively low correlation with the other two replicates in the CHG context (Pearson’s correlation, *r* = 0.76 and 0.77; Figure S3). After UV-B treatment, *R. apiculata* exhibited reduced levels of cytosine methylation in the CHG context. The extent of reduction varied greatly between replicates (Table 1, *F* test, p < 0.05), resulting in a slightly lower level of genome-wide CHG methylation for the treated plants (5.3% on average) than control (8.6% on average). In contrast, no significant change of methylation levels in all three contexts was found between UV-B-treated and control *A. marina* plants (Table 1).

**Table 1:** Methylation levels in *A. marina* and *R. apiculata* genome wide in three sequence contexts (CG, CHG and CHH, where H = A, T or C)

Species	Condition	Proportion of methylated sites			Genome-wide methylation level		
		CG	CHG	CHH	CG	CHG	CHH
*Avicennia marina*	Control	44.87 ± 0.73∗	28.95 ± 0.16	13.91±0.61	36.83 ± 2.61	23.15 ± 0.82	5.16 ± 0.33
	UV-B	45.36 ± 0.36	29.41 ± 0.33	14.54 ± 0.37	38.30 ± 1.58	24.77 ± 1.30	6.06 ± 0.54
*Rhizophora apiculata*	Control	24.41 ± 0.61	13.07 ± 0.48	5.94 ± 0.42	21.02 ± 1.37	8.63 ± 1.03	1.76 ± 0.20
	UV-B	24.44 ± 0.52	8.77 ± 5.63∗	4.75 ± 0.88	21.83 ± 0.57	5.28 ± 3.93	1.57 ± 0.26

The asterisks indicate the significant levels of variance in methylation levels across individuals between groups of UV-B treated and control.

∗ p < 0.05, *F* test.

**Figure 1:**
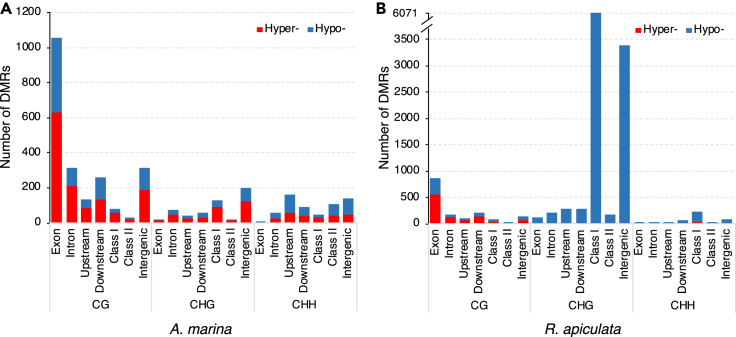
Genomic distribution of differentially methylated regions (DMRs) induced by UV-B exposure in *A. marina* and *R. apiculata* (A and B) The numbers of hyper- (red) and hypomethylated CG, CHG, and CHH DMRs (blue) associated with genes (including exon, intron, upstream and downstream sequences), transposable elements (including Class I and Class II elements) or other intergenic regions are shown separately for (A) *A. marina* and (B) *R. apiculata*.

Using a beta-binomial model (Feng et al., 2014), we identify differentially methylated regions (DMRs) between UV-B-treated and control plants for each species, including 2,175 CG, 518 CHG, and 591 CHH DMRs in *A. marina* and 1,520 CG, 10,495 CHG, and 380 CHH DMRs in *R. apiculata* (Figure S4). In *A. marina*, the extent of hyper- and hypomethylation induced by the UV-B treatment was largely comparable in all sequence contexts (Figure S4)*.* In *R. apiculata*, CHG and CHH DMRs were mostly hypomethylated while that of hyper-to hypo-methylation ration at CG DMRs was similar to *A. marina* (Figure S4). We then mapped the UV-B-induced DMRs to genomic and genic features of the *A. marina* and *R. apiculata* genomes. The vast majority of CG DMRs (80.8% in *A. marina* and 86.2% in *R. apiculata*) was near genes (in or within a 2-kb region upstream or downstream of the gene), predominately within exons (48.6% and 55.9%, respectively, Figure 1). CHG and CHH DMRs were distributed comparably across genomic features in *A. marina* (Figure 1A), whereas CHG (59.5%) and CHH (57.9%) DMRs in *R. apiculata* were almost entirely hypomethylated DMRs located in TEs (Class I or Class II transposons) (Figure 1B). In *R. apiculata*, CHG DMRs were enriched in *Copia* and unclassified LTR retrotransposons (Fisher’s exact test, both p < 0.001) while no enrichment on particular TE superfamilies was found for CHH DMRs (Fisher’s exact test, all p > 0.05). Both CHG and CHH DMRs in *R. apiculata* were preferentially located in long (>4 kb) TEs versus short TEs (Figure S5). Overall, high doses of UV-B radiation induced genome-wide remodeling of DNA methylation in the two mangrove species.

### Similarity and divergence in transcriptome changes of *A. marina* and *R. apiculata* under the UV-B treatment

**Figure 2:**
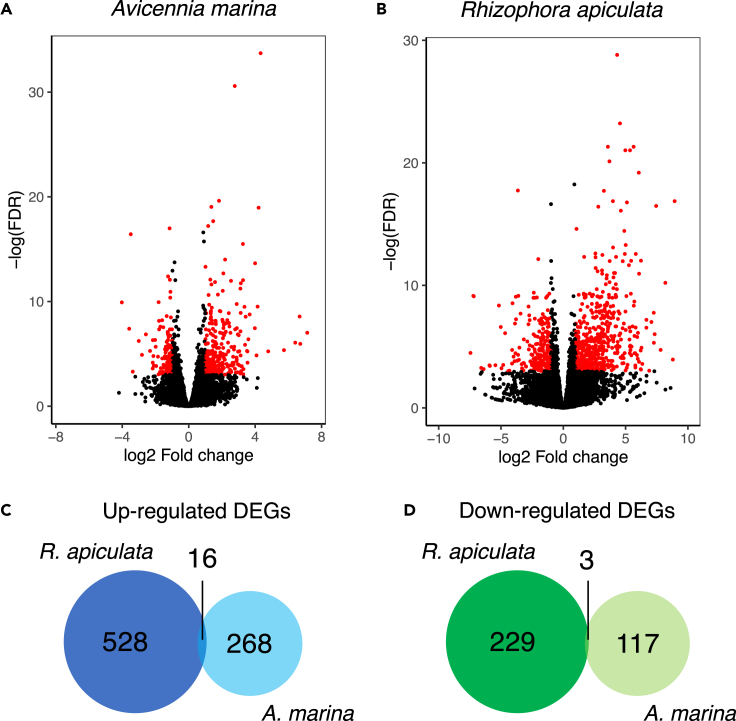
UV-B induced changes of gene expression in *A. marina* and *R. apiculata* (A and B) Volcano plot of differentially expressed genes (DEGs) between UV-B-treated and control plants of (A) *A. marina* and (B) *R. apiculata*. Red points represent DEGs with at least 2-fold change and adjusted p value (FDR) ≤ 0.05 between UV-B treated and control plants. (C and D) The numbers of up (orange) or downregulated (green) differentially expressed genes are indicated by up or downward arrows separately. Venn diagrams show the common (C) up and (D) downregulated differentially expressed orthologous genes between *A. marina* and *R. apiculata*.

To assess the potential impact of methylation changes on gene expression, we conducted RNA-seq using the samples described above. Expression levels were calculated as normalized counts in each species and genes with at least two-fold change and adjusted p value ≤0.05 between UV-B treated and control plants were considered as differentially expressed genes (DEGs). Principal component analysis (PCA) of the normalized count data separated the UV-B treated and control plants in both species, taking into account within-species gene expression variation (Figure S6A). Levels of gene expression between bio-replicates were highly correlated in *A. marina* (Pearson’s correlation, *r* = 0.75–0.94 for the control and *r* = 0.90–0.97 for the UV-B treated). The correlations were slightly lower in *R. apiculata* (Pearson’s correlation, *r* = 0.75–0.86 for the control and *r* = 0.43–0.92 for the UV-B treated) (Figure S6B). We identified 385 DEGs in *A. marina* and 757 in *R. apiculata.* We found almost two times as many upregulated as downregulated genes in both species (Figures 2A and 2B). Few DEGs were in common between *A. marina* and *R. apiculata*, including only 16 up-regulated and three down-regulated genes (Figures 2C and 2D). These results suggest that the two mangroves differ in their responses to UV-B radiation at the expression level.

**Figure 3:**
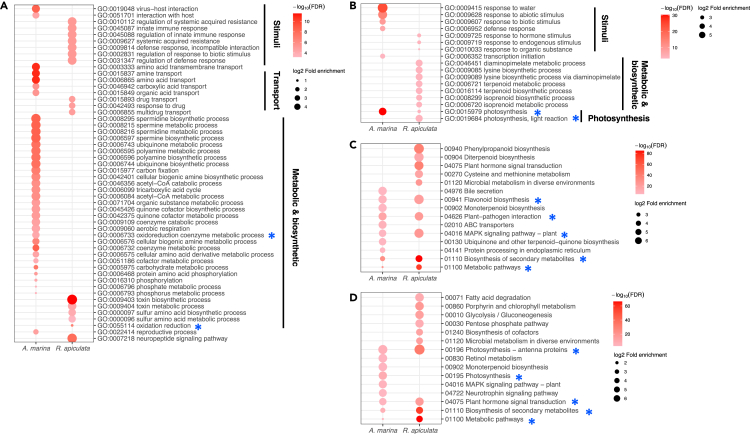
The gene ontology (GO) and Kyoto Encyclopedia of Genes and Genomes (KEGG) pathway enrichments of the differentially expressed genes (DEGs) between UV-B treated and control *A. marina* and *R. apiculata* plants. (A and B) GO terms enriched in the upregulated and (B) downregulated genes between UV-B treated and control *A. marina* and *R. apiculata*. Blue asterisks indicate GO terms shared between the two mangrove species. (C and D) Top ten KEGG pathways enriched in the upregulated and (D) downregulated genes between UV-B treated and control *A. marina* and *R. apiculata*. Blue asterisks indicate KEGG pathways that are identical between the two mangrove species.

Gene Ontology (GO) analyses revealed that *A. marina* has more GO terms overrepresented (Chi-square test, FDR ≤0.05) among the up-regulated genes (36 terms) but fewer GO terms (Chi-square test, FDR ≤0.05) among the down-regulated genes (6 terms) than *R. apiculata* (16 and 12 terms for up-regulation and down-regulation respectively; Figure 3 and Table S2). In both species, the GO categories over-represented in the UV-B-induced DEGs were associated with responses to biotic and abiotic stimuli as well as diverse metabolic and biosynthetic processes (Figure 3; Table S2), supporting the view that high doses of UV-B stimulate nonspecific signal transduction pathways that are involved in the response to various stresses (Jenkins, 2009; Wargent and Jordan, 2013). Furthermore, the over-representative GOs share identical or similar functions between species, including oxidation reduction for the upregulated DEGs (“oxidoreduction coenzyme metabolic process” (GO:0006733) in *A. marina* and “oxidation reduction” (GO:0055114) in *R. apiculata*; Table S2), as well as photosynthesis for the downregulated DEGs (“photosynthesis” (GO:0015979) in both *A. marina* and *R. apiculata*, and “photosynthesis, light reaction” (GO:0019684) in *R. apiculata*; Table S2). These results are consistent with the idea that plants face enhanced oxidative stress and inhibit photosynthesis under elevated UV-B radiation (Ruhland et al., 2005; Yannarelli et al., 2006).

Using the Kyoto Encyclopedia of Genes and Genomes (KEGG) analyses of DEGs, we further dissected the similarity and divergence of transcriptome changes in *A. marina* and *R. apiculta* in response to the UV-B treatment (Figure 3; Table S3). In both species, the highlighted pathways significantly overrepresented in the upregulated and downregulated DEGs were “flavonoid biosynthesis” (KO: 00941) and “photosynthesis” (KO: 00195 or KO: 00196), respectively (Table S3). Among the 14 genes involved in flavonoid biosynthesis, six were significantly upregulated by UV-B in *R. apiculata,* whereas only two genes on top of the pathway (*phenylalanine ammonia-lyase*, *PAL* and *chalcone synthase*, *CHS*; also up-regulated in *R. apiculata*) showed increased expression in *A. marina* (Table S4; Figure S7). Among photosynthetic pathways, ten of 12 genes of the *light-harvesting chlorophyll protein complex* (*LHC*) were dramatically repressed by UV-B in *R. apiculata* while only two (*LHCA1* and *LHCB4*) of these components were down-regulated in *A. marina* (Table S4). The two species also have distinct KEGG pathways enriched among upregulated genes, including “ABC transporters” (KO: 00130), “Ubiquinone and other terpenoid-quinone biosynthesis” (KO: 00130), “Bile secretion” (KO: 04141), etc. in *A. marina*, and “Phenylpropanoid biosynthesis” (KO: 00940), “Cysteine and methionine metabolism” (KO: 00270), “Diterpenoid biosynthesis” (KO: 00904), etc. in *R. apiculata* (Figure 3; Table S3).

These results indicate that *A. marina* and *R. apiculata* share some transcriptional responses but also activate distinct pathways against UV-B irradiation. Moreover, the *A. marina* transcriptome changes less than *R. apiculata* after exposure to enhanced UV-B radiation. This effect cannot be explained by the random noise caused by experimental reproducibility (Figure S6).

### UV-B-induced CG methylation changes are uncoupled from differential gene expression

**Figure 4:**
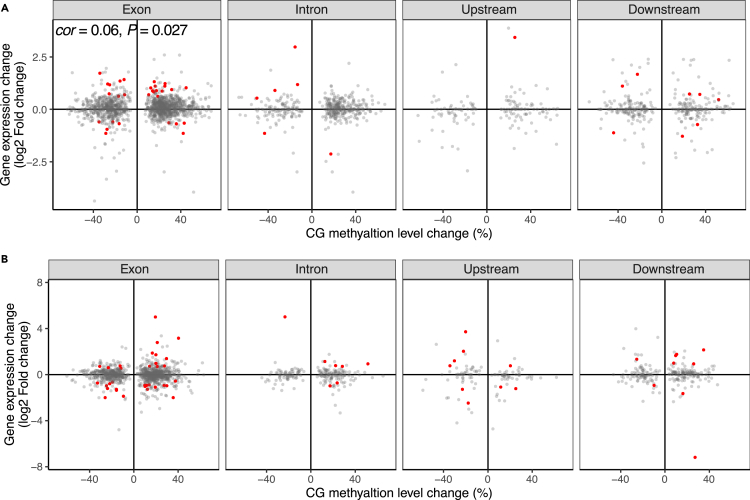
Association betweeen methylation changes of DMRs and expression changes of DMR-associcated genes under UV-B treatment (A and B) Quadrant plot of methylation changes of the UV-B-induced differentially methylated regions (DMRs) and expression changes of the DMR-associated differentially expressed genes in (A) *A. marina* and (B) *R. apiculata*. The x axis represents the CG methylation difference of DMRs in the UV-B treated plants relative to control. The y axis indicates the expression difference (log2-fold change) of the DMR-associated genes in the UV-B treated plants relative to control. The red points indicate genes with significant (Benjamini–Hochberg FDR ≤ 0.05) differential expression. *cor*, Pearson's correlation coefficient; p, p value. Only significant (p ≤ 0.05) correlation coefficients were shown.

To evaluate the potential impact of UV-B-induced methylation changes on gene expression, we examined expression of genes that are associated with UV-B-induced CG DMRs in both mangrove species. A gene was considered CG-DMR associated if at least one CG-DMR was located inside the gene or within 2-kb upstream or downstream of it. In *A. marina*, 17 of the 1,758 CG-DMR-associated genes (1.0%) were identified as DEGs. A similar proportion was found in *R. apiculata* (1.8%, 23 of 1,310). In both species, more than half of the CG-DMR-associated DEGs contained CG DMRs in their exons (Figure 4). The correlation between methylation changes of CG DMRs and expression changes of their associated genes was significant only for genes containing CG DMRs in their exons in *A. marina* (Pearson’s correlation, *r* = 0.06, p < 0.05). No significant correlation was detected between the up- or down-regulation of DEGs and the hyper- or hypomethylation of associated DMRs in either species (Figure 4). The UV-B induced DEGs were not overrepresented among CG-DMR-associated genes relative to the whole transcriptome in *A. marina* or *R. apiculata* (Fisher’s exact test, both p > 0.05). Therefore, UV-B-induced CG methylation changes have a negligible impact, if any, on genome-wide gene expression in *R. apiculata*.

### UV-B-induced DNA hypomethylation is associated with transcriptional reactivation of TEs in *R. apiculata*

**Figure 5:**
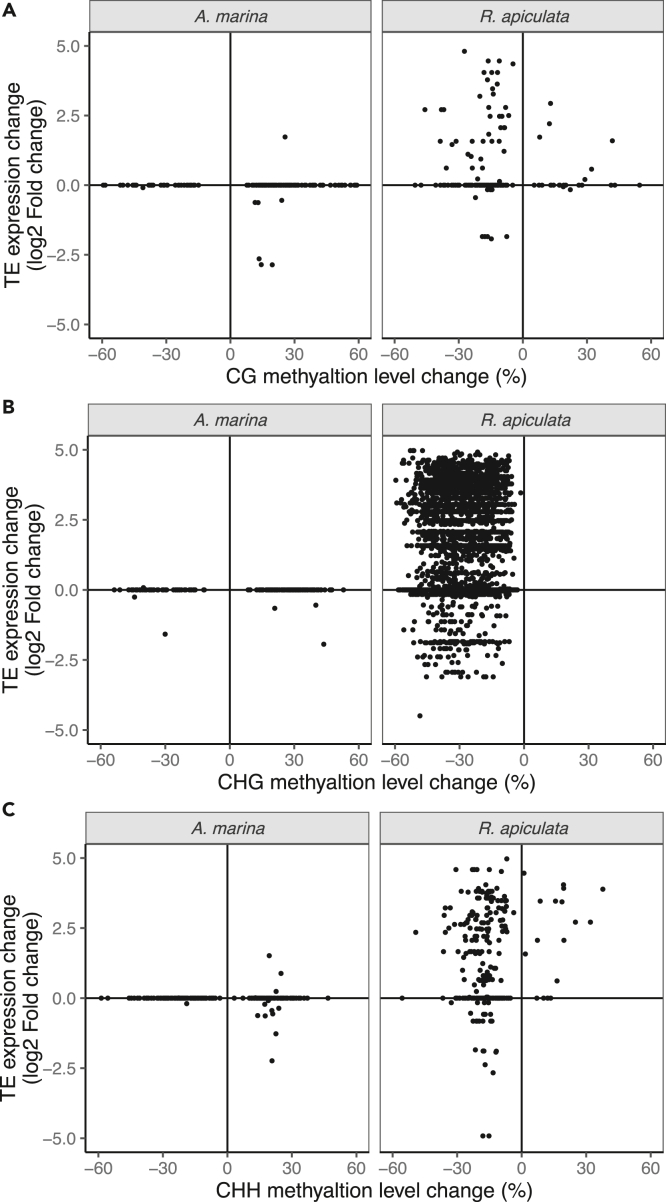
Association between methylation changes of the UV-B-induced differentially methylated regions (DMRs) and expression changes of DMR-associated transposable elements (TEs) (A–C) Quadrant plot of the CG (A), CHG (B) and CHH (C) methylation changes of the UV-B-induced differentially methylated regions (DMRs) and expression changes of the DMR-associated differentially expressed genes in *A. marina* and *R. apiculata.* The x axis represents the DNA methylation difference among DMRs in different sequence contexts in the UV-B treated plants relative to control. The y axis indicates the expression difference (log2-fold change) of the DMR-associated TEs in the UV-B treated plants relative to control.

We next examined the impact of UV-B-induced methylation changes on TE expression, given that non-CG DMRs are clustered in TEs of both mangroves (Figure 1). We considered TEs overlapping defined DMRs by at least 1 bp as DMR-associated and the corresponding DMRs are referred to as TE-associated DMRs hereafter. In *A. marina*, few DMR-associated TEs altered their expression in response to UV-B treatment, and most of these exhibited decreased expression levels and were associated with hypermethylated DMRs (Figures 5A–5C). The pattern is consistent and largely comparable for the DMR-associated TEs in all three sequence contexts of *A. marina* (Figures 5A–5C). In contrast, a large fraction of the DMR-associated TEs in *R. apiculata* are upregulated and associated with hypomethylated DMRs after the UV-B treatment (Figures 5A–5C). This pattern is most prominent in TEs associated with the CHG-DMRs (Figure 5B), followed by CHH- and CG-DMRs (Figures 5C and 5A). Downregulations of TEs were less frequent in *R. apiculata,* whether TEs are associated with hyper- or hypo-methylation (Figures 5A–5C). More than half of the CHG- (55.4%) and CHH-DMR-associated TEs (53.6%) in *R. apiculata* come from RapLTR06. RapLTR06 is the largest LTR retrotransposon family in *R. apiculata* (Wang et al., 2018), comprising 80.7% and 70.3% of the upregulated CHG- and CHH-DMR-associated TE copies, respectively (Figure S8). This result is consistent with the observation that *R. apiculata* has more young to middle aged (0–4 Myrs) retrotransposons compared to *A. marina* (Figure S9).

TEs often have negative impact on the expression of neighboring genes through epigenetic effects (Hollister and Gaut, 2009). Considering the association between TEs and the UV-B-induced hypomethylated DMRs in *R. apiculata*, we expected that genes flanking TE-associated-DMRs might exhibit increased expression after UV-B exposure in this species. Among the 310 genes containing or flanking (within 2-kb upstream or downstream) TE-associated DMRs in *R. apiculata*, six were identified as DEGs in the UV-B treated plants relative to control (Table S5). We see more upregulations (4) than downregulations (2) and the majority of these DEGs (5/6) were associated with CHG-DMRs (Table S5). These DEGs include *nudix hydrolase 4,* involved in plant detoxification processes in response to abiotic and biotic stresses (Yoshimura and Shigeoka, 2015), *cysteine-rich receptor-like protein kinase (CRK) 10* playing vital roles in plant disease resistance and cell death (Quezada et al., 2019), *glutamate decarboxylase (GAD) 4* required for normal oxidative stress tolerance (Coleman et al., 2001), *transcription factor MYB30* regulating oxidative and heat stress responses (Liao et al., 2017), *late embryogenesis abundant protein At1g64065-like* with major role in drought and other abiotic stresses tolerance in plants (Magwanga et al., 2018), and *uncharacterized protein LOC110654808 isoform X2* (Table S5).

UV-B-induced DNA hypomethylation is tightly associated with transcriptional reactivation of TEs. Although TE de-repression is coincident with upregulation of genes involved in stress tolerance, the overall impact of TE de-repression on the expression of TE-flanking genes is limited in *R. apiculata* and even less in *A. marina*.

### Deficiency of small interfering RNAs (siRNAs) is associated with non-CG hypomethylation of TEs in *R. apiculata*

**Figure 6:**
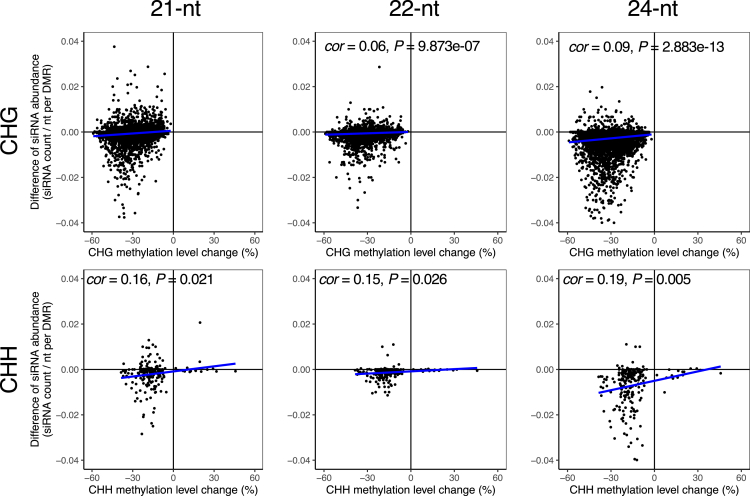
Correlation analyses of UV-B induced changes of siRNA abundance and non-CG (CHG and CHH) methylation level of transposable elements (TEs)-associated differentially methylated regions (DMRs) in *R. apiculata*. The x axis represents the changes of methylation level in the TE-associated DMRs in UV-B treated *R. apiculata* plants relative to control. The y axis represents changes of siRNA (21-, 22- and 24-nt) abundance in the TE-associated DMRs in UV-B treated *R. apiculata* plants relative to control. The blue lines indicate regression curves. *cor*, Pearson's correlation coefficient; p, p value. Only significant (p < 0.05) correlation coefficients are shown.

SiRNAs direct *de novo* methylation of cytosine in the CHH context and reinforce DNA methylation in the CHG context through the RNA-directed DNA methylation (RdDM) pathway (Tamiru et al., 2018; Erdmann and Picard, 2020). Using small RNA-seq, we examined the potential association between DNA methylation and siRNA expression in response to the UV-B treatment in *A. marina* and *R. apiculata*. siRNA abundance was calculated as read counts per base pair for each DMR. In *R. apiculata*, abundance of the 21-, 22- and 24-nt siRNAs mapping to the TE-associated non-CG DMRs was dramatically decreased in UV-B treated plants relative to control, particularly for the 24-nt siRNAs (Figure 6). Pairwise comparisons of UV-B-induced changes of siRNA abundance and methylation level on the TE-associated CHG or CHH DMRs detected weak but significant positive correlations in all pairs (Pearson’s correlation, *r* = 0.06 to 0.19, all p < 0.05) except for the pair between the 21-nt siRNA abundance and the level of CHG methylation (Figure 6). In *A. marina*, siRNAs were rare in TE-associated-DMRs and showed little variation in abundance in response to UV-B treatment (Figure S10). UV-B-induced changes of the 24-nt siRNA abundance positively correlated with changes of the CHH methylation levels for the TE-associated CHH DMRs in *A. marina* but the correlation was not significant (Pearson’s correlation, *r* = 0.14, p > 0.05, Figure S10). These results suggest that UV-B induced TE hypomethylation is associated with reduction of siRNA abundance in both mangrove species.

## Discussion

Plant responses to UV-B radiation have been receiving increasing attention (Frohnmeyer and Staiger, 2003; Fina et al., 2017; Jiang et al., 2021), partially due to ozone depletion and the expected increase in UV radiation at the earth's surface (Austin and Wilson, 2006; Bornman et al., 2019). However, little is known about the mechanisms by which tropical plants may ameliorate the detrimental effects of constant high UV-B radiation. Such knowledge will provide a comprehensive understanding of how plants adapt to environmental stress in the era of climate change. Using genome-wide DNA methylation analysis, we show that high levels of UV-B treatment induce dramatic non-CG hypomethylation preferentially in TEs in *R. apiculata* but not in *A. marina* (Figure 1). Consistently, gene mis-expression is more widespread in *R. apiculata* than in *A. marina* (Figure 2), although relatively few genes change expression in either of these mangrove species compared with temperate plants (Mucha et al., 2015; Fina et al., 2017; Zhao et al., 2017). While the lack of stress symptoms under UV-B treatment confirmed that both *A. marina* and *R. apiculata* are well adapted to UV-B radiation, the epigenetic and transcriptional analyses suggest that the molecular mechanisms underlying their UV-B adaptation might be different.

At the epigenetic level, *A. marina* showed great genome stability under UV-B treatment whereas *R. apiculata* exhibited predominantly non-CG hypomethylation together with massive de-repression of TEs just like *Arabidopsis* (Jiang et al., 2021). The UV-B-induced TE reactivation in *R. apiculata* is consistent with the genome shock hypothesis (McClintock, 1984). It is thought that stress-induced reactivation of TEs can help facilitate plant adaptation to extreme environments by either increasing genetic diversity or alternating gene regulatory networks (Almojil et al., 2021; Srikant and Drost, 2020). Although we cannot directly measure the mutational effects of TE reactivation in *R. apiculata*, we did observe that the relaxation of TE epigenetic regulation is associated with the up-regulation of TE-adjacent loci. Most of these genes are known to participate in various stress responsive processes (Table S5). Nevertheless, the overall impact of TE de-repression on flanking gene expression is limited in *R. apiculata*, although some particular TE-associated genes may play auxiliary roles in the cross-talk between UV-B and other stimuli signaling through the mitogen-activated protein kinase (MAPK) cascade.

Why the two mangrove species show different epigenetic responses under the same UV-B treatment is enigmatic. One possibility is that genes involved in the UV-B perception and/or DNA methylation pathways are expressed differentially between species. One such candidate is UVR8, a UV-B receptor recently reported to inhibit activity of DRM2 in RdDM (RNA-directed DNA methylation) pathway causing TE hypomethylation and reactivation under elevated UV-B radiation in *Arabidopsis* (Jiang et al., 2021). However, the expression levels of *UVR8* and *DRM2* remained unchanged in both *A. marina* and *R. apiculata* after UV-B treatment (Table S4). Among other genes in the DNA methylation and demethylation pathways, only *KTF1* (*kow domain-containing transcription factor 1*) was up-regulated in *A. marina* under UV-B treatment (Table S4). KTF1 functions as an adaptor protein that binds scaffold transcripts generated by Pol V (RNA polymerase V) and recruits AGO4 (Argonaute 4) and AGO4-bound siRNAs to form RdDM effector complexes for TE silencing (He et al., 2009). The upregulation of *KTF1* in *A. marina* might contribute to increased epigenetic control of TEs by siRNAs. Consistent with this speculation, there are significantly more hyper- than hypomethylated non-CG DMRs in Class I TEs compared with the whole genome of *A. marina* (χ^2^ test, p < 0.001, Figure 1). Nevertheless, this speculation still needs to be verified by further experiments. Interestingly, enhanced TE repression in *A. marina* under UV-B treatment echoes the greater demand for TE repression in *A. marina* than *R. apiculata* in nature, as the former contains a higher proportion of TEs (31.4%) than the latter (17.1%). We previously found that a fraction of TEs in *R. apiculata* have the potential to be activated by stress, which might increase genetic diversity and thus evolutionary potential of *R. apiculta* to adapt to extreme intertidal environments (Wang et al., 2018). Stringent control of TEs in *A. marina* in contrast to the relaxation of TE repression in *R. apiuclata* may represent the need to balance genome integrity and variability under UV-B exposure, depending on the host genome constitution.

At the expressional level, *A. marina* also showed greater resistance to UV-B than *R. apiculata*. First, there are fewer differentially expressed genes in *A. marina* under UV-B exposure (Figure 2). A homeostatic transcriptome may be indicative of mangrove tolerance to environmental stress considering their long-term adaptation (Liang et al., 2012). Particularly, although both species down-regulate *light-harvesting chlorophyll protein complex* (*LHC*) genes of PSII and upregulate genes participating in the flavonoid biosynthesis pathway under UV-B treatment, much fewer mis-expressed genes in these functional categories are found in *A. marina* than in *R. apiculata* (Table S4). LHC proteins are involved in photosynthetic pathways and vulnerable to UV-B radiation (Sztatelman et al., 2015). Together with unchanged levels of chlorophyll a and chlorophyll b contents under UV-B treatment (Figures S2A and S2B), lack of down-regulation of *LHC* genes suggests that *A. marina* does not suffer from the inhibition of photosynthesis that is usually observed under UV-B stress (Xie et al., 2020). The production of flavonoids (crucial protective compounds against UV-B; Tsuda, 2012) can be induced by excess UV light (Del Valle et al., 2020; Zoratti et al., 2014). Rare upregulation of flavonoid biosynthesis genes under UV-B in *A. marina* is probably associated with the extremely high level of total flavonoids in this species (∼43.8 mg/g; Figure S2C), whether treated with high dose UV-B or not, in comparison with that in *R. apiculata* (∼6.8 mg/g; Figure S2C) or *Arabidopsis* (∼0.4 mg/g) (Wang et al., 2016). Second, *A. marina* specifically up-regulates genes involved in ATP-binding cassette (ABC) transporter biosynthesis (Tables S3 and S4). This pathway participates directly in the active transport of a wide range of molecules across membranes (Geisler and Murphy, 2006). ABC transporters are thought to be the first line of cellular defense against chemical or physical stress. They may be involved in photo-protection by modulating the epidermal concentration of UV-absorptive secondary metabolites, such as flavonoids in plants (Li et al., 1993). In sea urchins, ABC transporters ABCB1 and ABCC1 were reported to protect gametes and embryonic cells against the harmful effects of UV-B (Leite et al., 2014). Third, *A. marina* up-regulates genes involved in ubiquinone and other terpenoid-quinone biosynthesis (Tables S3 and S4). This can increase the generation of phylloquinone (vitamin K1) which is required for the stability of the PSI complex involved in photosynthesis (Wang et al., 2017). Overall, *A. marina* differs from *R. apiculata* in responses against UV-B radiation, which are characterized by the active transport of secondary metabolites across membranes and the protection of the photosynthesis system, although both species share the same photoprotective response of accumulating UV-absorbing compounds such as flavonoids and anthocyanin.

In conclusion, our results demonstrate that mangrove species show different sensitivity to UV-B in both epigenetic and gene expression responses. While UV-B-induced TE de-repression is common and thus requires stringent epigenetic regulation to maintain genome integrity, transcriptional changes underlying UV-B defense mechanisms can be different between species. Our results point towards the key genes and pathways that may contribute to the success of UV-adaptation of tropical forest plants.

### Limitations of the study

It should be noted that the UV fluorescent lamp used (ranging from 280 to 360 nm with a peak at 306 nm, Model G15T8E, Sankyo-Denki, Japan) produces both UV-B and UV-A light. The observed epigenetic and expression responses of mangroves under treatment may be partially attributable to UV-A exposure. Moreover, this may also lead to an overestimation of UV-B levels under experimental conditions as the UV-B spectrum radiometer we used (UV-313, Beijing Normal University Photoelectric Instruments Factory) measures UV light from 290 to 340 nm with a peak at 313 nm. However, such bias won’t change our conclusion that mangroves have well adapted to high UV because *Arabidopsis* under the same experimental conditions suffered from severe leaf discoloration and died after three days of treatment (Figure S11).

## STAR★Methods

### Key resources table

**Table:** 

REAGENT or RESOURCE	SOURCE	IDENTIFIER
**Chemicals, peptides, and recombinant proteins**		

CTAB	Sigma	Cat#H6269
EDTA	Sigma	Cat#E6758
Tris	Sigma	Cat#V900312
2-Mercaptoethanol	ThermoFisher	Cat#21985023
acetone	ThermoFisher	Cat#T_702A060015
anhydrous ethanol	ThermoFisher	Cat#E/0550DF/15

**Critical commercial assays**		

Micro Plant Flavonoids Assay Kit	Solarbio	Cat#BC1330
Zymo EZ DNA Methylation-Gold Kit	Zymo Research	Cat#D5005
EpiGnome™ Kit	Epicenter	Cat#EGMK81312

**Deposited data**		

Raw data generated in this study	This study	GenBank: PRJNA704509

**Software and algorithms**		

Trimmomatic (v.0.36)	Bolger et al. (2014)	https://github.com/usadellab/Trimmomatic
Bismark (v.0.22.1)	Krueger and Andrews (2011)	https://github.com/FelixKrueger/Bismark/releases/tag/0.22.1
methylKit (v.1.17.4)	Akalin et al., 2012	https://github.com/al2na/methylKit
HISAT2 (v2.1.0)	Kim et al. (2019)	https://github.com/DaehwanKimLab/hisat2/releases/tag/v2.1.0
HTSeq (v.0.12.4)	Anders et al. (2015)	https://github.com/htseq/htseq/releases/tag/release_0.12.4
DESeq2 (v.1.26.0)	Love et al. (2014)	https://bioconductor.org/packages/release/bioc/html/DESeq2.html
OrthoMCL (v.2.0.9)	Li et al. (2003)	https://orthomcl.org/orthomcl/app
agriGO (v.2.0)	Tian et al., 2017	http://bioinfo.cau.edu.cn/agriGO/
Bowtie (v.1.1.2)	Langmead et al. (2009)	https://sourceforge.net/projects/bowtie-bio/files/bowtie/1.1.2/
R (v.3.6.2)	GNU project	https://www.R-project.org

### Resource availability

#### Lead contact

Further information and requests for resources and reagents should be directed to and will be fulfilled by the lead contact, Prof. Tian Tang (lsstt@mail.sysu.edu.cn).

#### Materials availability

This study did not generate new unique reagents.

### Experimental model and subject details

#### Plant materials and growth conditions

Propagules of *A. marina* and *R. apiculata* were collected from Qinlan Harbor, Hainan, China (19° 37'N, 110° 47'N) and cultivated in a greenhouse under a natural photoperiod with the daily maximum UV-B radiation ranging from 0.9 to 1.4 μW/cm^2^ (UV-313, Beijing Normal University Photoelectric Instruments Factory, detection range from 290 to 340 nm with a peak at 313 nm). For each species, seedlings with more than four true leaves (usually about 30 cm tall) were used for the UV-B treatment following a previous method (Ma et al., 2016). For the UV-B treatment, seedlings were exposed to additional UV-B radiation from one UV fluorescent lamp (ranging from 280 to 360 nm with a peak at 306 nm, Model G15T8E, Sankyo-Denki, Japan) for 8 h/day (from 10:00 to 18:00) in a light incubator for 7 days, with an average fluency rate of 92.6 μW/cm^2^ (UV-313, Beijing Normal University Photoelectric Instruments Factory) at a mean distance of 35 cm to plants. In parallel, control seedlings were exposed to white light at 1812-1816 lux intensity (Pro’sKit MT-4617LED, Prokit’s Industries Co., Ltd.) delivered by LEDs (FSL YZ15, Foshan Electrical and Lighting Co., Ltd) in another light incubator to avoid possible effects of diurnal or circadian rhythms. After treatments, all plants were moved back to the greenhouse every day and cultivated under a natural photoperiod as described above. Three independent biological replicates were performed under each condition (UV-B treated vs. control) for both *A. marina* and *R. apiculata*.

### Method details

#### Morphological and physiological analyses

For morphological analysis, we randomly selected one leaf at a similar height from each of the three biological replicates and took pictures of theses leaves *in situ* every day before and during the UV-B treatment (day 0 to day 7) for *A. marina* and *R. apiculata*. We also measured chlorophyll a, chlorophyll b, and total flavonoid content in leaves of UV-B treated and control plants for each species after the UV-B treatment. Fresh clean leaf samples (0.1 g) were sliced and incubated in 15 mL of pigment extraction solution containing acetone and anhydrous ethanol (1:1, v/v) in the dark for 24 h at 25°C. Chlorophyll content was determined as described by Arnon (1949). Total flavonoids were determined using the Micro Plant Flavonoids Assay Kit (Solarbio, Beijing, China) following the manufacturers’ instructions. Rutin was used to make a standard calibration curve. All measurements were carried out in triplicate for each of the three independent biological replicates.

### Quantification and statistical analysis

#### Bisulfite sequencing and analyses

Fresh young leaves of *A. marina* and *R. apiculata* were sampled from the UV-B stressed and unstressed seedlings separately. Genomic DNA and total RNA were immediately extracted from the same sample using a modified CTAB protocol (Yang et al., 2008). Genomic DNA were bisulfite converted using the Zymo EZ DNA Methylation-Gold Kit (Zymo Research, Orange, CA, USA) and purified to prepare whole-genome bisulfite sequencing libraries with the EpiGnome™ Kit (Epicenter, Madison, WI, USA) following the manufacturers’ instructions. All libraries were sequenced on an Illumina HiSeq 4000 platform (Illumina, San Diego, CA, USA) and 150 bp paired-end reads were harvested with Q30 quality control.

Raw reads were trimmed and filtered to remove adapters or low-quality bases using Trimmomatic v.0.36 (Bolger et al., 2014). Clean reads were mapped to the *A. marina* or *R. apiculata* genomes (He et al., 2020) using Bismark v.0.22.1 (Krueger and Andrews, 2011) with default parameters. Only uniquely mapping reads were used for subsequent methylation analyses. Bisulfite conversion efficiency was calculated from the proportion of unconverted Cs in all methylation contexts together (CG, CHG and CHH, where H is A, T, or C) from the lambda (Promega D1521) genome. Bisulfite conversion efficiency was then used as the expected probability in a binomial test to determine cytosines that were either methylated (false discovery rate, FDR ≤ 0.05) or not followed by Benjamini-Hochberg multiple test correction (Benjamini and Hochberg, 1995). Only cytosines covered by more than five sequencing reads were considered in the following study. Methylation level was determined by calculating the proportion of methylated cytosines among total cytosines by methylation context (Schultz et al., 2012). To inspect the reproducibility between biological replicates, sliding window analysis (window size =100 kb and step size =50 kb) of methylation levels was conducted for each sample in all three sequence contexts. Pearson’s correlation between methylation levels in pairs of biological replicates was estimated within either the UV-B treated or control group for each sequence context in each species.

Differentially methylated regions (DMRs) were identified using methylKit v.1.17.4 (Akalin et al., 2012) with a beta-binomial model (Feng et al., 2014) followed by Benjamini–Hochberg multiple test correction (Benjamini and Hochberg, 1995). A tilling window approach was used in methylKit with window size of 100 bp and step size of 50 bp. All 100 bp tiles were called differentially methylated between the experimental and reference group if the corrected P values met a given threshold (FDR ≤ 0.05) alongside a minimum number of Cs (five Cs) and a minimum fold change of 0.3 as described by Dubin et al., 2015. Adjacent tiles identified as DMRs were collapsed into a single tile.

#### RNA-seq and analyses

Total RNA extracted as mentioned above was used for RNA-seq on Illumina HiSeq 4000 to generate 150 bp paired-end reads and analyzed as previously described (Wang et al., 2018). After quality control, clean reads were mapped to the appropriate genomes using HISAT2 v2.1.0 (Kim et al., 2019) with default parameters. Raw reads mapped to each gene were analyzed using HTSeq v.0.12.4 (Anders et al., 2015) with the parameter: -s no, considering only uniquely mapped reads. Reads mapped to each transposable element (TE) were counted in parallel except that multi mapping reads on TEs were retained and weighted by the number of hits. Expression levels of genes or TEs were calculated as normalized counts using DESeq2 v.1.26.0 (Love et al., 2014). Differentially expressed genes (DEGs) or TEs were determined by DESeq2 v.1.26.0 (Love et al., 2014) requiring FDR ≤ 0.05 and ≥ 2 fold change. Principal component analysis (PCA) were conducted for each species using a regularized log2 transform of the normalized counts of all genes as generated by DESeq2 v.1.26.0 (Love et al., 2014). Pearson correlation of gene expression (log2 of the normalized counts) between biological replicates was calculated within the UV-B treated or control group of each species using R v.3.6.2 (https://www.R-project.org).

TE annotation of *R. apiculata* was adopted from a previous study (Wang et al., 2018) and the TEs of *A. marina* were identified using the same procedure. We measured the distance from a TE to its nearest neighboring gene, including both 2-kb upstream and downstream genes, as described previously (Wicker et al., 2016). Orthologous gene clusters between *A. marina* and *R. apiculata* were constructed by OrthoMCL v.2.0.9 (Li et al., 2003) with pipelines and parameters adopted previously (Xu et al., 2017). Statistical analyses were conducted using R v.3.6.2.

#### GO and KEGG analyses

Gene Ontology (GO) terms and Kyoto Encyclopedia of Genes and Genomes (KEGG) annotations of *A. marina* and *R. apiculata* unigenes were obtained from previously published study (Xu et al., 2017). GO term enrichment analyses were carried out using agriGO v.2.0 (Tian et al., 2017) with a Chi-square test followed by Benjamini-Hochberg correction (Benjamini and Hochberg, 1995). Significance level was set as FDR ≤ 0.05. On the basis of the KEGG annotation, we used KEGG Mapper (Kanehisa and Sato, 2020) to reconstruct target KO (KEGG Orthology) terms into pathways and carried out Fisher’s exact test combined with Benjamini-Hochberg correction (Benjamini and Hochberg, 1995) to test for statistical significance (FDR ≤ 0.05) of specific pathway enrichment.

#### Small RNA sequencing and analyses

Total RNA was used for small RNA library construction and sequencing as described previously (Wen et al., 2016). Sequencing reads were quality controlled and filtered for structural non-coding RNAs, including ribosomal RNA (rRNA), transfer RNA (tRNA), small nuclear RNA (snRNA), small nucleolar RNA (snoRNA), known microRNAs (miRNAs), and reads outside 18- to 30-nt as described previously (Wang et al., 2018). The remaining putative small interfering RNA (siRNAs) were aligned to the *A. marina* or *R. apiculata* genomes using Bowtie v.1.1.2 (Langmead et al., 2009) with no mismatch allowed. Expression levels of the 21-, 22-, or 24-nt siRNAs at each DMR were calculated as read counts per base pair per DMR. Multiple mapping siRNAs were weighted by the number of hits they produced. Correlation between siRNA expression and methylation changes of DMRs was estimated using R v.3.6.2.

## Data Availability

The sequencing data has been deposited at the GenBank data libraries and are publicly available as of the date of publication. Accession numbers are listed in the Key resources table. This paper does not report original code. Any additional information required to reanalyze the data reported in this paper is available from the lead contact upon request.

## Supplemental information

Document S1. Figures S1–S11 and Table S1

Table S2. GO annotation of the differentially expressed genes induced by UV-B stress, related to Figure 3

Table S3. Top ten KEGG pathways that are significantly enriched in the UV-B-induced differentially expressed genes (DEGs), related to Figure 3

Table S4. Expression profiles of genes involved in the UV-B related pathways log2FC, logarithm of fold change ratios of genes that are upregulated (red), downregulated (blue) and unchanged (black) in response to UV-B stress, related to Figure 3.

Table S5. Differentially expressed genes containing or flanking TE-associated DMRs in *R. apiculata* log2FC, logarithm of fold change ratios of genes or transposable elements (TEs) that are up or downregulated in response to UV-B stress. adjust-P, adjusted P values for the expression difference, related to Figure 5.
