# Supplementary material for: Epigenetic and transcriptional responses underlying mangrove adaptation to UV-B
Source: iScience. 2021 Sep 20;24(10):103148. doi: 10.1016/j.isci.2021.103148 (PMC8496181; doi:10.1016/j.isci.2021.103148)
Supplement: Document S1. Figures S1–S11 and Table S1 [file mmc1.pdf]

iScience, Volume *24*

Supplemental information

Epigenetic and transcriptional responses underlying mangrove adaptation to UV-B

Yushuai Wang, Chenglong Huang, Weishun Zeng, Tianyuan Zhang, Cairong Zhong, Shulin Deng, and Tian Tang

Supplemental Figures

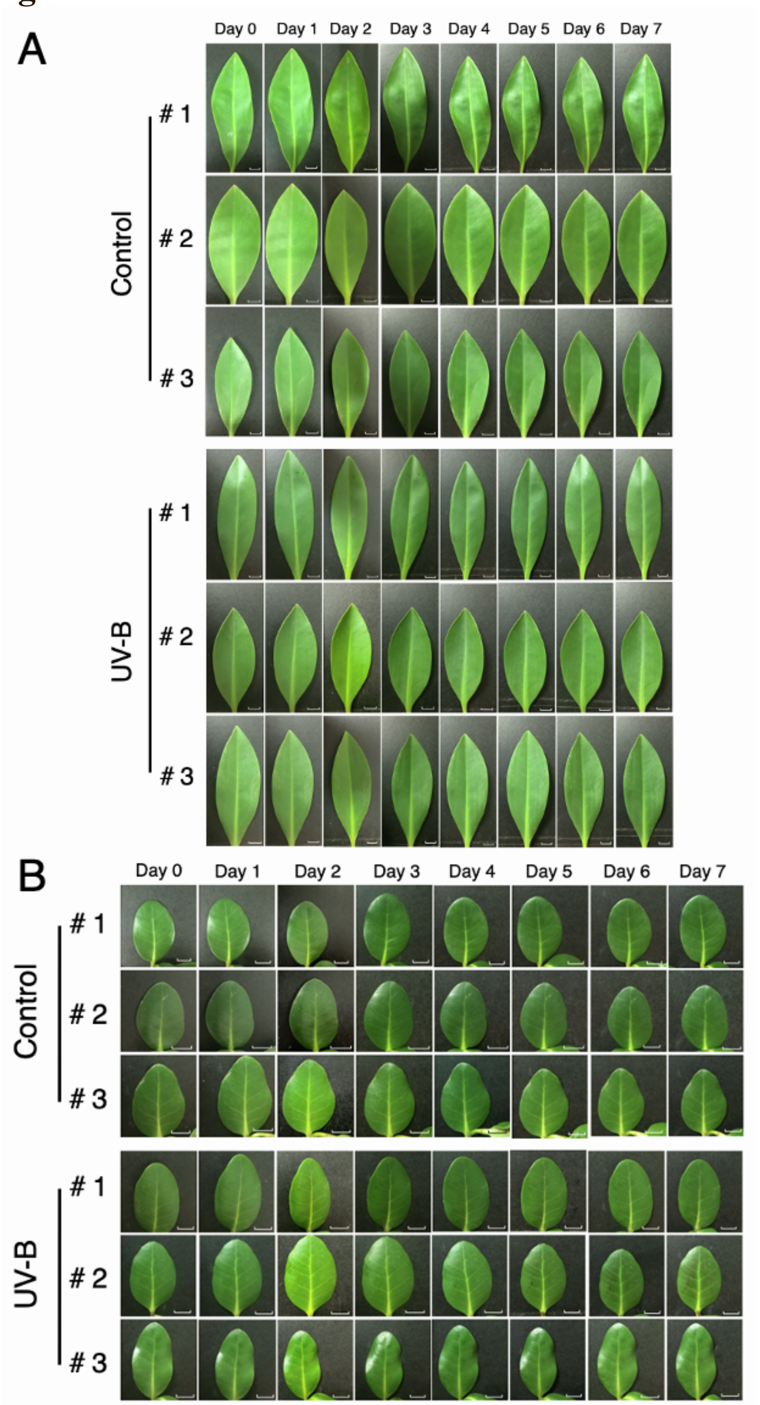

Figure S1. Photographs of leaves of UV-B treated and control plants of (A) *Avicennia marina* and (B) *Rhizophora apiculata*. The UV-B treatment was conducted for seedlings of *A. marina* and *R. apiculata* with three biological replicates (#1-3) for seven days. Randomly-selected leaves each from a similar height of a biological replicate were imaged *in situ* before (Day 0) and during the UV-B treatment (Day1-7). The scale indicates 1 cm. Notice that a leaf of the UV-B treated replicate #2 of *A. marina* had a scratch before the UV-B treatment and got darker during the treatment. *[Related to Figure 1 and Figure 2]*

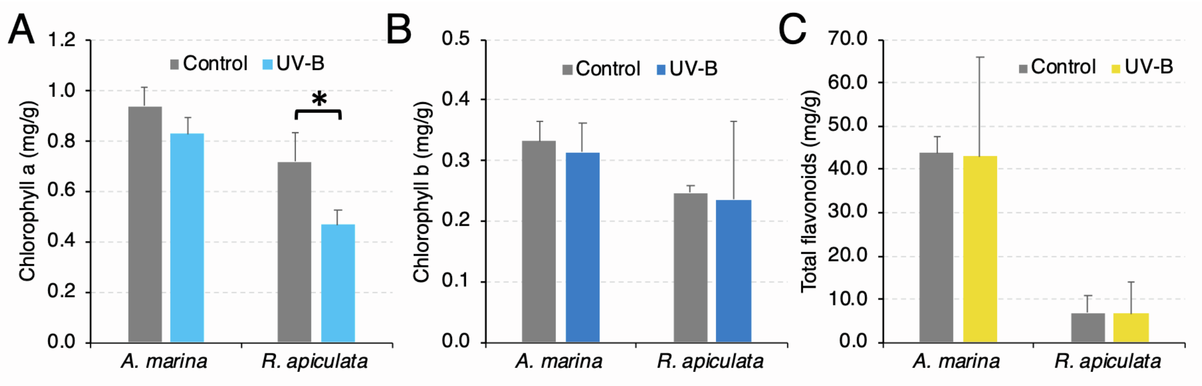

Figure S2. Chlorophyll a, chlorophyll b, and flavonoid levels in UV-B treated and control plants of *Avicennia marina* and *Rhizophora apiculata*. Data are presented as mean ± SD (n = 3). Asterisk indicates a significant difference between UV-B treated and control plants at $P < 0.05$, by two-tailed *t*-test. *[Related to Figure 1 and Figure 2]*

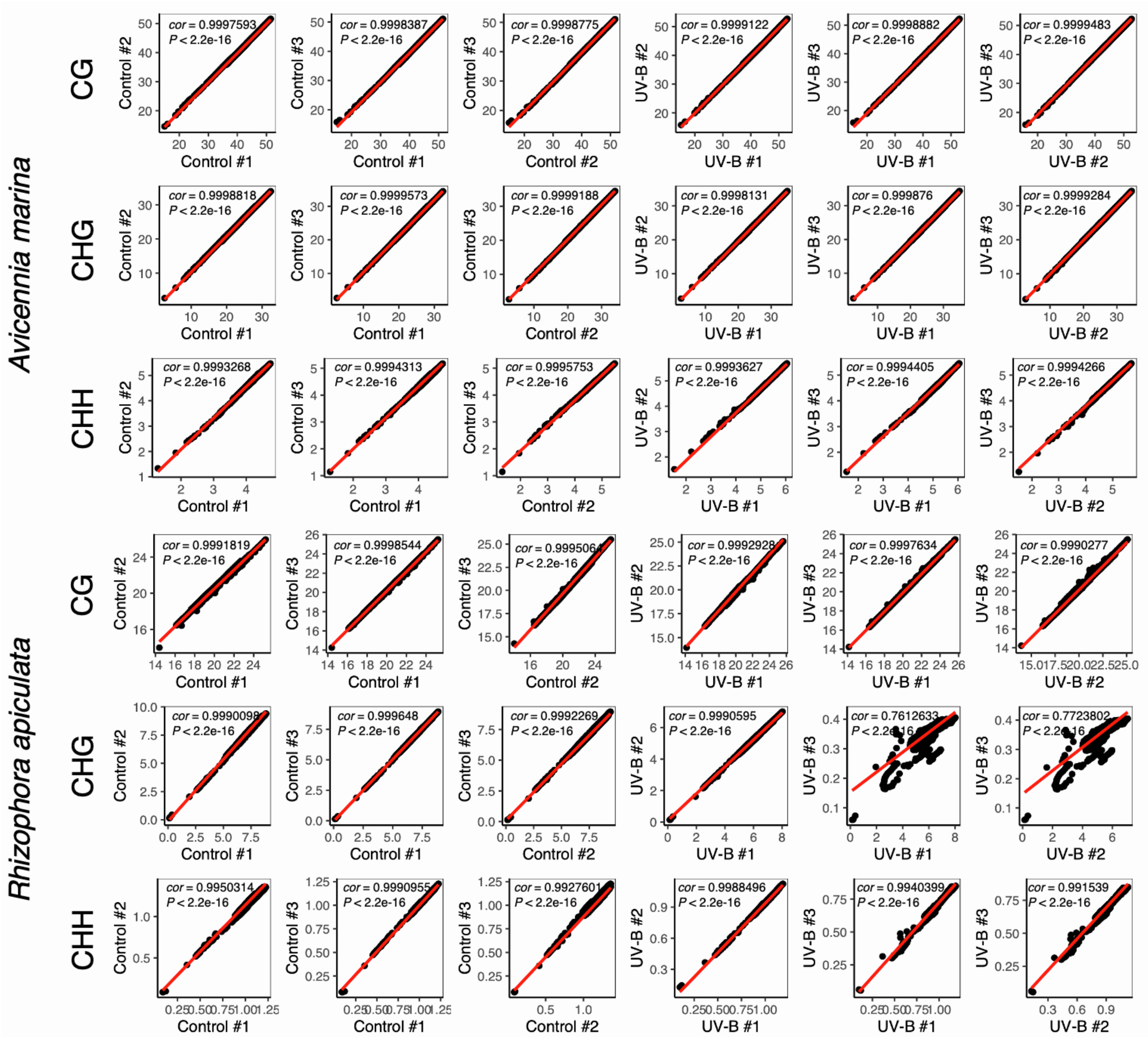

Figure S3. Pairwise comparison of DNA methylation levels between biological replicates within the UV-B treated or control group of *A. marina* and *R. apiculata*. Sliding window analysis of methylation levels in all sequence contexts (CG, CHG and CHH) was conducted with window size 100 kb and step size 50 kb. The methylation levels of the 100-kb windows were used to calculate Pearson's correlation coefficient between replicates within group. *[Related to Figure 1]*

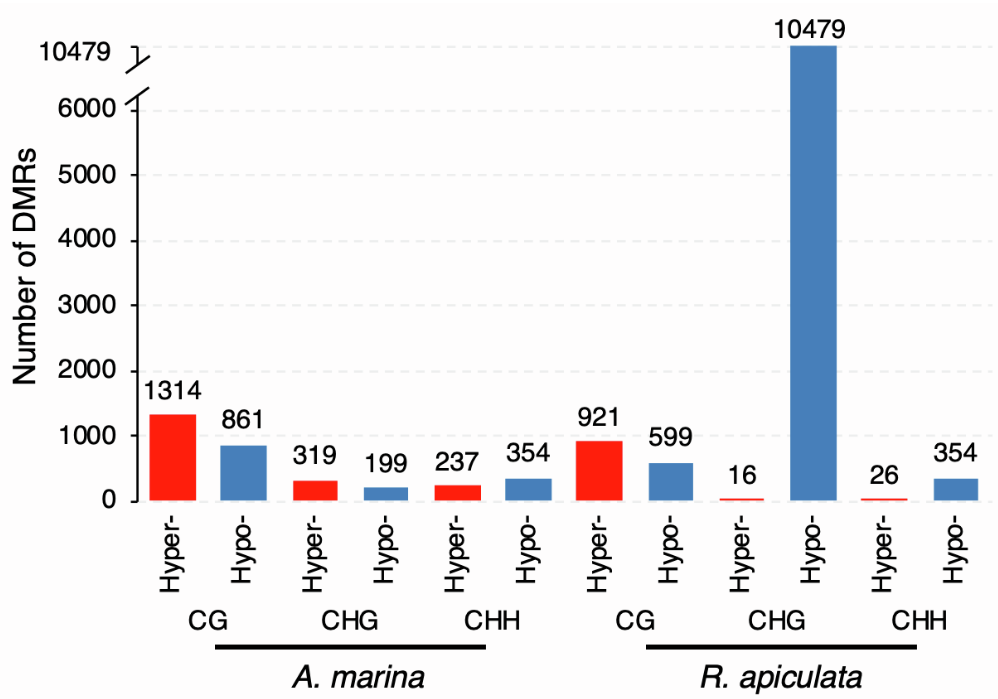

Figure S4. Frequency distribution of differentially methylated regions (DMRs) induced by UV-B exposure in *A. marina* and *R. apiculata*. All DMRs were classified into three categories according to their sequence context of CG, CHG or CHH. Hyper- and hypo-methylated are indicated in red and blue, respectively. *[Related to Figure 1]*

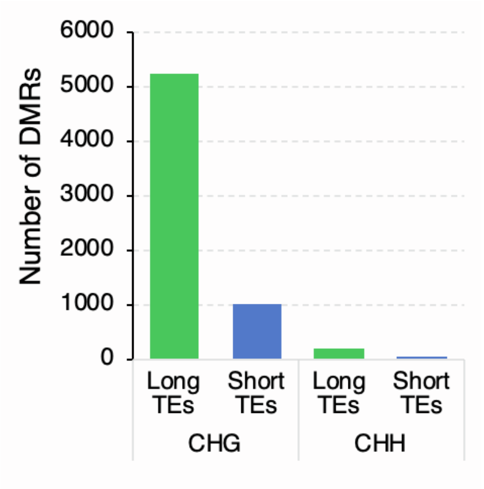

Figure S5. Distribution of non-CG DMRs on long (> 4kb) and short (<= 4kb) transposable elements (TEs). *[Related to Figure 5]*

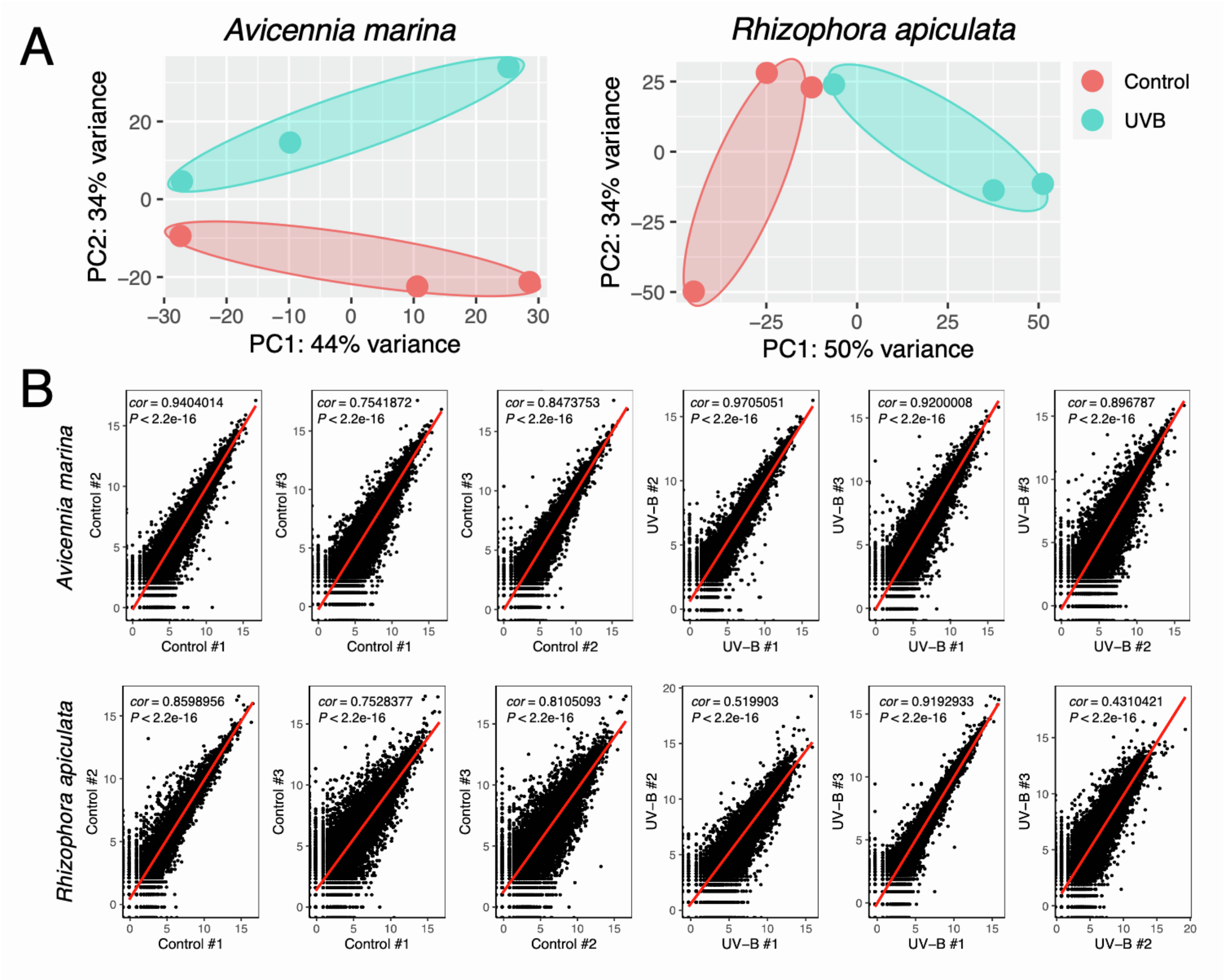

Figure S6. Reproducibility of gene expression across the UV-B treated and control plants of *Avicennia marina* and *Rhizophora apiculata*. (A) Principal component analysis (PCA) plots of transcriptome data from the UV-B treated and control plants of *A. marina* and *R. apiculata*. The expression levels of all genes were used for each species. (B) Pearson correlation of gene expression profiles between biological replicates within either the UV-B treated or control group of *A. marina* and *R. apiculata*. Gene expression levels were calculated as log2 normalized count as generated by DEseq2. *[Related to Figure 2]*

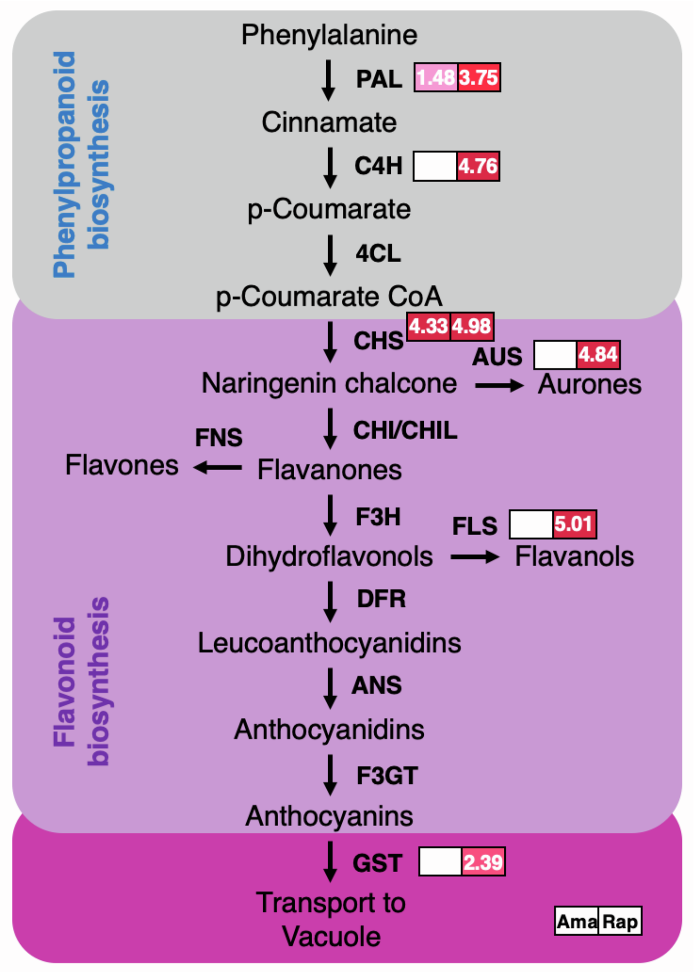

Figure S7. UV-B-induced differential expression of flavonoid biosynthesis genes in *A. marina* and *R. apiculta*. Gene list and the flavonoid biosynthesis pathway are adapted from Davies et al., (2020). Heatmap shows log2 fold changes of gene expression in UV-B treated plants relative to control. Only genes that were differentially expressed in at least one species are listed. The insignificant expression change was indicated in white while significant expression changes were indicated in different red colors. Numbers indicate log2 fold changes of gene expression after UV-B treatment. Ama, *A. marina*; Rap, *R. apiculta*. *PAL*, *phenylalanine ammonia-lyase*; *C4H*, *cinnamate 4-hydroxylase*; *4CL*, *4-coumarate:CoA ligase*; *CHS*, *chalcone synthase*; *CHI*, *chalcone isomerase*; *CHIL*, *chalcone isomerase-like*; *F3H*, *flavanone 3-hydroxylase*; *DFR*, *dihydroflavonol 4-reductase*; *ANS*, *anthocyanidin synthase*; *F3GT*, *flavonoid 3-O-glucosyltransferase*; *AUS*, *aureusidin/aurone synthase*; *FNS*, *flavone synthase*; *FLS*, *flavonol synthase*; *GST*, *glutathione S-transferase*. *[Related to Figure 3]*

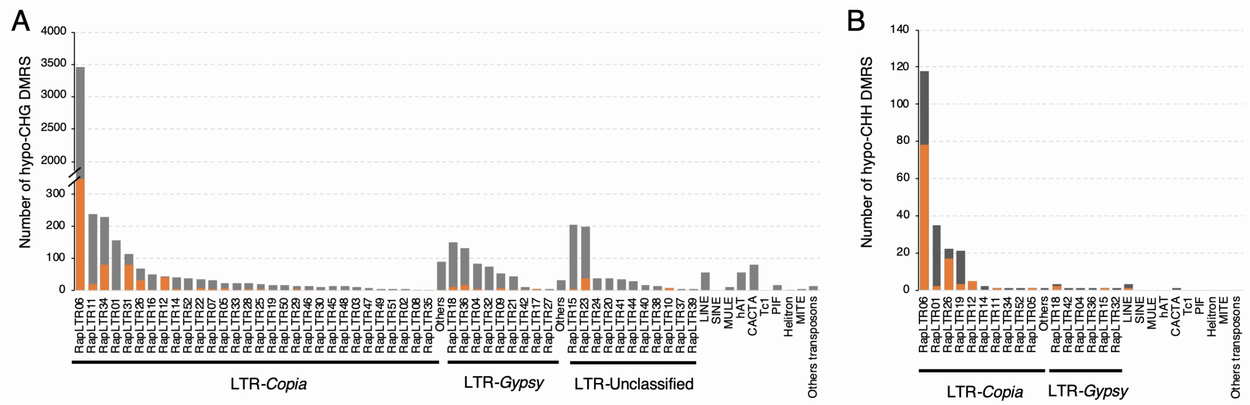

Figure S8. Frequency distribution of hypomethylated (A) CHG- and (B) CHH-DMRs that are associated with transposable elements (TEs) in *R. apiculata*. Gray bars indicate the total number of DMRs that overlap individual TE families. Orange bars indicate the number of DMRs associated with expressed TE copies for individual TE families. LTR, long-terminal repeat. *[Related to Figure 5]*

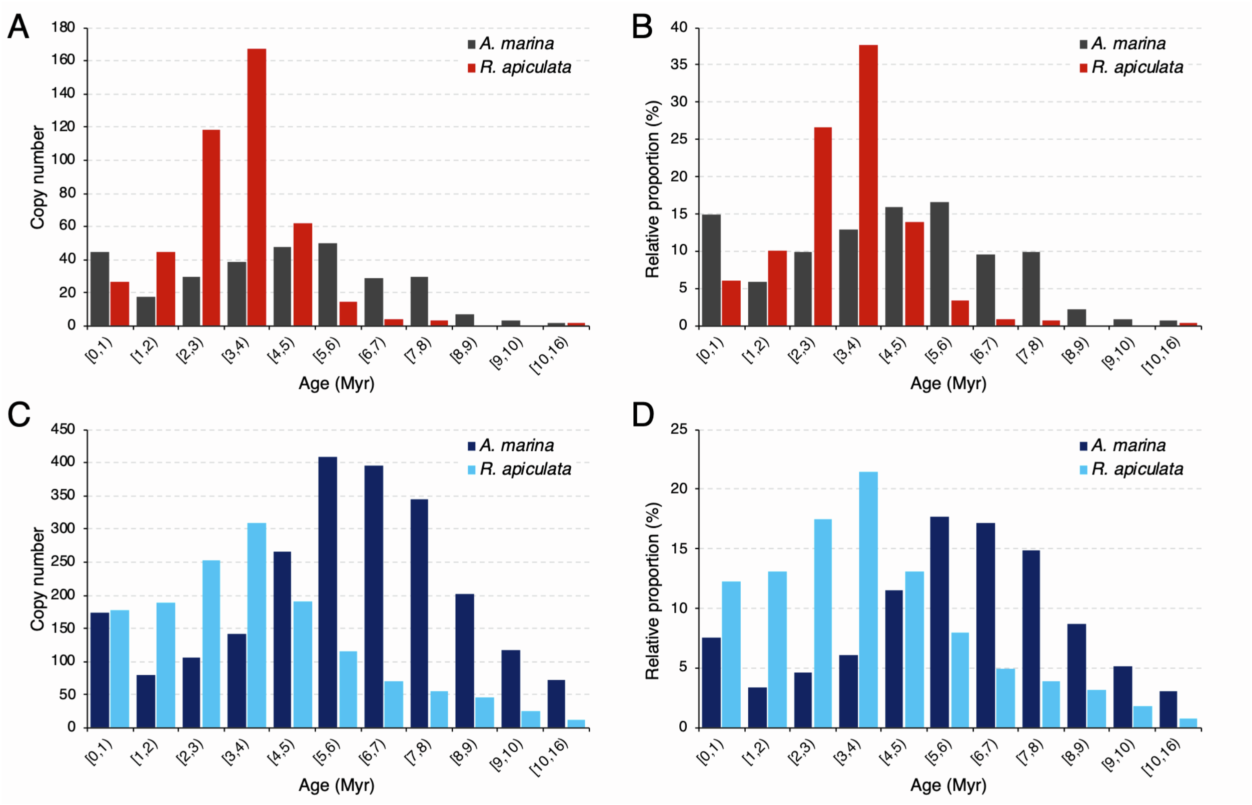

Figure S9. Age distribution of LTR retrotransposons in *Avicennia marina* and *Rhizophora apiculata*. (A) Copy number of the largest LTR retrotransposon family, (B) relative proportion of the largest LTR retrotransposon family, (C) copy number of all intact LTR retrotransposons, (D) relative proportion of all intact LTR retrotransposons within each age bin were shown separately. The age of each LTR retrotransposon was calculated assuming a molecular clock as described previously by Ma and Bennetzen (2004). *[Related to Figure 5]*

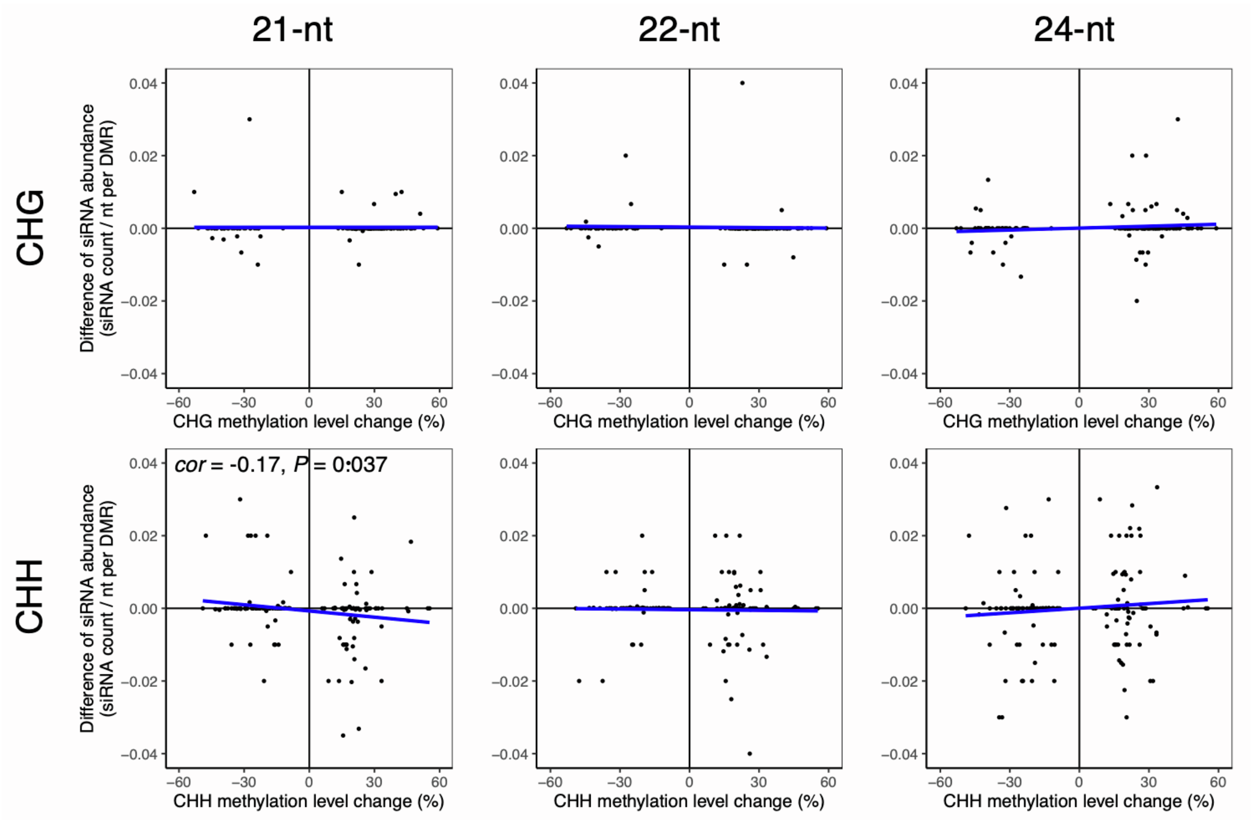

Figure S10. Correlation analyses of UV-B induced change in siRNA abundance and non-CG (CHG and CHH) methylation level of transposable elements (TEs)-associated differentially methylated regions (DMRs) in *A. marina*. The x-axis represents methylation level change in the TE-associated DMRs in UV-B treated *A. marina* plants relative to control. The y-axis represents changes of siRNA (21-, 22- and 24-nt) abundance in the TE-associated DMRs in UV-B treated *A. marina* plants relative to control. The blue lines indicate regression curves for Pearson's correlation. *cor*, Pearson's correlation coefficient; *P*, *P* value. Only significant ($P < 0.05$) correlation coefficients were shown. *[Related to Figure 6]*

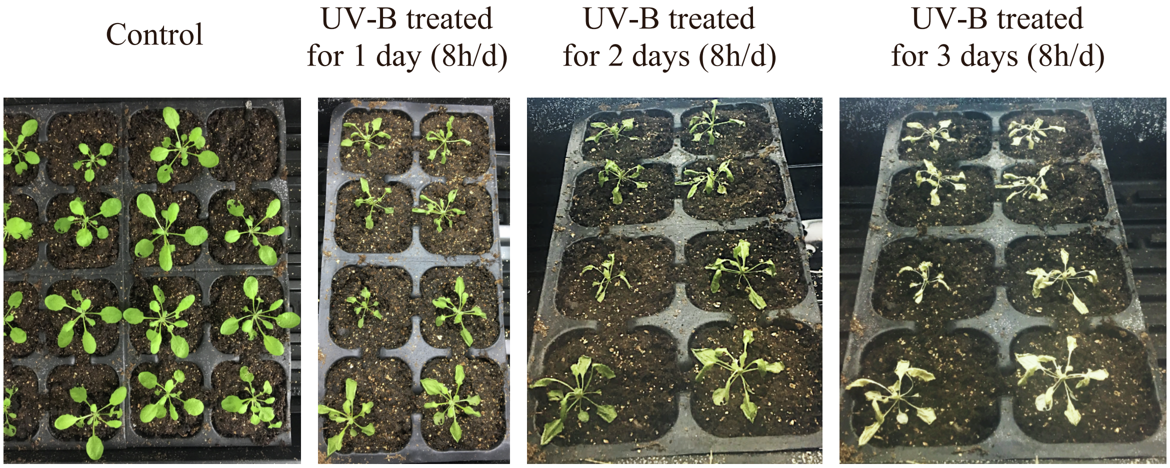

Figure S11. *Arabidopsis* under UV-B treatments for different durations. The same experimental conditions as for mangroves were used. *[Related to Figure 1 and Figure 2]*

Supplemental Tables

Table S1. Summary statistics of whole genome bisulfite sequencing data. *[Related to Figure 1]*

Species	Condition	Sample ID	Conversion rate (%)	# of unique mapped reads	Mapping efficiency	Sequencing depth	Coverage of C sites		
							CG	CHG	CHH
Avicennia marina	Control	Repetition #1	99.53	65,570,068	88.10%	17.95	88.61	90.46	88.16
		Repetition #2	99.58	64,996,146	87.70%	18.09	87.88	89.59	85.37
		Repetition #3	99.57	64,557,031	87.30%	17.19	88.08	90.01	85.76
	UV-B	Repetition #1	99.55	64,605,701	87.00%	14.26	79.47	80.94	72.12
		Repetition #2	99.59	63,890,406	86.10%	14.14	82.47	84.05	76.89
		Repetition #3	99.56	64,900,053	87.10%	17.72	85.36	87.27	81.28
Rhizophora apiculata	Control	Repetition #1	99.61	56,021,766	75.40%	30.48	92.02	93.30	91.72
		Repetition #2	99.60	55,212,983	74.50%	30.14	91.58	92.86	90.24
		Repetition #3	99.58	56,875,498	76.60%	29.76	92.47	93.69	92.36
	UV-B	Repetition #1	99.54	56,072,844	75.10%	24.74	91.10	92.37	89.98
		Repetition #2	99.61	56,950,806	76.80%	30.82	92.15	93.47	91.83
		Repetition #3	99.60	55,017,732	74.10%	28.95	91.09	92.25	90.16
